# Pangloss: A Tool for Pan-Genome Analysis of Microbial Eukaryotes

**DOI:** 10.3390/genes10070521

**Published:** 2019-07-10

**Authors:** Charley G. P. McCarthy, David A. Fitzpatrick

**Affiliations:** 1 Genome Evolution Laboratory, Department of Biology, Maynooth University, W23 F2K8 Maynooth, Ireland; 2 Human Health Research Institute, Maynooth University, W23 F2K8 Maynooth, Ireland

**Keywords:** pangenomes, bioinformatics, microbial eukaryotes, fungi

## Abstract

Although the pan-genome concept originated in prokaryote genomics, an increasing number of eukaryote species pan-genomes have also been analysed. However, there is a relative lack of software intended for eukaryote pan-genome analysis compared to that available for prokaryotes. In a previous study, we analysed the pan-genomes of four model fungi with a computational pipeline that constructed pan-genomes using the synteny-dependent Pan-genome Ortholog Clustering Tool (PanOCT) approach. Here, we present a modified and improved version of that pipeline which we have called Pangloss. Pangloss can perform gene prediction for a set of genomes from a given species that the user provides, constructs and optionally refines a species pan-genome from that set using PanOCT, and can perform various functional characterisation and visualisation analyses of species pan-genome data. To demonstrate Pangloss’s capabilities, we constructed and analysed a species pan-genome for the oleaginous yeast *Yarrowia lipolytica* and also reconstructed a previously-published species pan-genome for the opportunistic respiratory pathogen *Aspergillus fumigatus*. Pangloss is implemented in Python, Perl and R and is freely available under an open source GPLv3 licence via GitHub.

## 1. Introduction

Species pan-genomes have been extensively studied in prokaryotes, where pan-genome evolution is primarily driven by rampant horizontal gene transfer (HGT) [1,2,3,4]. Pan-genome evolution in prokaryotes can also vary substantially as a result of lifestyle and environmental factors; opportunistic pathogens such as *Pseudomonas aeruginosa* have large “open” pan-genomes with large proportions of accessory genes, whereas obligate intracellular parasites such as *Chlamydia* species have smaller “closed” pan-genomes with larger proportions of conserved core genes and a smaller pool of novel genetic content [5,6,7]. Studies of pan-genome evolution within eukaryotes has not been as extensive as that of prokaryotes to date, as eukaryote genomes are generally more difficult to sequence and assemble in large numbers relative to prokaryote genomes. However, consistent evidence for pan-genomic structure within eukaryotes has been demonstrated in plants, fungi and plankton [8,9,10,11,12]. Unlike prokaryote pan-genomes, eukaryote pan-genomes evolve via a variety of processes besides HGT, these include variations in ploidy and heterozygosity within plants [8], and cases of introgression, gene duplication and repeat-induced point mutation in fungi and plankton [9,10,11,12].

The majority of software and pipelines available for pan-genome analysis are explicitly or implicitly intended for prokaryote datasets. For example, the commonly-cited pipeline Roary is intended for use with genomic location data generated by the prokaryote genome annotation software Prokka [13,14]. A number of other methodologies such as seq-seq-pan or SplitMEM use genome alignment or de Bruijn graph-based approaches for pan-genome construction, which are usually computationally impracticable for eukaryote analysis [15,16]. Other common pan-genome methodologies, such as the Large Scale BLAST Score Ratio (LS-BSR) approach or the Markov Cluster Algorithm (MCL)/MultiParanoid-dependent Pan-genome Analysis Pipeline (PGAP), may have potential application in eukaryote pan-genome analysis but as of writing no such application has occurred [17,18,19,20]. Of the eukaryote pan-genome analyses in the literature, some construct pan-genomes by mapping and aligning sequence reads using pipelines such as the Eukaryotic Pan-genome Analysis Toolkit (EUPAN) [8,12,21], or have constructed and characterised eukaryote pan-genomes using bespoke BLAST-dependent or clustering algorithm-dependent sequence clustering approaches [9,10,12]. In a previous article, we constructed and analysed the species pan-genomes of four model fungi including *Saccharomyces cerevisiae,* using the synteny-based Pan-genome Ortholog Clustering Tool (PanOCT, https://sourceforge.net/projects/panoct/) method in addition to our own prediction and analysis pipelines [11,22]. PanOCT was initially developed for prokaryote pan-genome analysis, and constructs a pan-genome from a given dataset by clustering homologous sequences from different input genomes together into clusters of syntenic orthologs based on a measurement of local syntenic conservation between these sequences, referred to as a conserved gene neighbourhood (CGN) score, and BLAST score ratio (BSR) assessment of sequence similarity [22,23]. Crucially, this synteny-based approach allows PanOCT to distinguish between paralogous sequences within the same genome when assessing orthologous sequences between genomes [11].

Here, we present a refined and improved version of our PanOCT-based pan-genome analysis pipeline which we have called Pangloss. Pangloss incorporates reference-based and ab initio gene model prediction methods, and synteny-based pan-genome construction using PanOCT with an optional refinement based on reciprocal sequence similarity between clusters of syntenic orthologs. Pangloss can also perform a number of downstream characterisation analyses of eukaryote pan-genomes, including Gene Ontology (GO-slim) term enrichment in core and accessory genomes, selection analyses in core and accessory genomes and visualisation of pan-genomic data. To demonstrate the pipeline’s capabilities we have constructed and analysed a species pan-genome for the oleaginous yeast *Yarrowia lipolytica* using Pangloss [24]. *Y. lipolytica* is one of the earliest-diverging yeasts and has seen various applications as a non-conventional yeast model for protein secretion, regulation of dimorphism and lipid accumulation, and is a potential alternative source for biofuels and other oleochemicals [25,26,27,28,29,30,31]. We have also reconstructed the species pan-genome of the opportunistic respiratory pathogen *Aspergillus fumigatus* from a previous study as a control [11]. Pangloss is implemented in Python, Perl and R, and is freely available under an open source GPLv3 licence from http://github.com/chmccarthy/Pangloss.

## 2. Materials and Methods

### 2.1. Implementation

**Figure 1:**
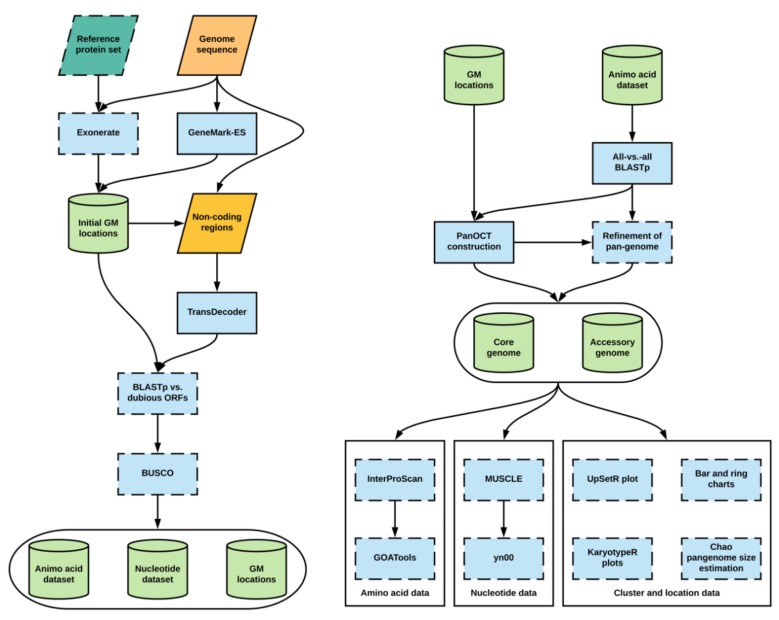
Workflow of Pangloss. Optional analyses represented with dotted borders. Refer to implementation for further information. GM: Gene model.

Pangloss is predominantly written in Python with some R and Perl components, and is compatible with macOS and Linux operating systems. Pangloss performs a series of gene prediction, gene annotation and functional analyses to characterise the pan-genomes of microbial eukaryotes. These analyses can be enabled by the user by invoking their corresponding flags on the command line, and many of the parameters of these analyses are controlled by Pangloss using a configuration file. The various dependencies for eukaryote pan-genome analysis using Pangloss are given in Table 1 along with versions tested and the workflow of Pangloss is given in Figure 1, both are described in greater detail below [32,33,34,35,36,37,38,39,40,41,42,43,44,45]. A user manual as well as further installation instructions and download locations for all dependencies of Pangloss are available from http://github.com/chmccarthy/Pangloss/.

#### 2.1.1. Gene Model Prediction and Annotation

By default, Pangloss performs its own gene model prediction to generate nucleotide and protein sequence data for all gene models from each genome in a dataset (Figure 1). Pangloss also generates a set of PanOCT-compatible gene model location data for each genome. Gene model prediction can be skipped by including the argument --no_pred at the command-line if such data has already been generated, or the user can solely run gene model prediction with no downstream analysis by including the argument --pred_only at the command-line. For each genome in a dataset, up to three methods of prediction are used:

1. All predicted protein sequences from a user-provided reference genome are queried against each genome using Exonerate (https://www.ebi.ac.uk/about/vertebrate-genomics/software/exonerate), with a heuristic protein2genome search model [33]. Translated gene model top-hits with an alignment score of ≥90% of the maximum possible alignment score as determined by Exonerate are retained as potential gene models. This search step is parallelized through Python’s multiprocessing library and can be optionally disabled by the user by including the argument --no_exonerate at the command-line.
2. Ab initio hidden Markov model (HMM)-dependent gene model prediction is performed using GeneMark-ES (http://exon.gatech.edu/GeneMark/) with self-training enabled [38]. If the species of interest is fungal, the user can enable a fungal-specific branch point site prediction model in the configuration file. If the user has also predicted gene models via step 1, those gene models whose locations do not overlap with gene models predicted via GeneMark-ES are incorporated into the latter dataset.
3. All remaining non-coding regions of the genome are extracted and subjected to position weight matrix (PWM)-dependent gene model prediction using TransDecoder (https://github.com/TransDecoder/TransDecoder/wiki) [39]. Any remaining predicted gene models with a length of ≥200 amino acids are included in the final gene model dataset.

There are a number of optional steps after that the user can take to assess the quality of gene model prediction within a dataset (Figure 1). The user can filter gene model sets for potential pseudogenes by querying a set of known dubious genes (either user-curated or from an appropriate resource such as the *Saccharomyces* Genome Database) against each gene model set using BLASTp (enabled via the --qc command-line argument) [46,47]. Any gene models whose top BLASTp hit against a dubious gene has sequence coverage of ≥70% are removed from further analysis. The completeness of each gene model set can also be assessed using BUSCO (https://gitlab.com/ezlab/busco) (enabled via the --busco command-line argument), with the appropriate dataset assigned by the user [41].

#### 2.1.2. BLASTp and PanOCT Analysis

By default, all predicted gene models within a dataset are combined and an all-vs.-all BLASTp search is performed within Pangloss with a user-defined e-value cut-off (default = 10^−4^) (Figure 1). However, if the user prefers to perform the all-vs.-all BLASTp step on their own high-performance computational environment they can skip the search via the --no_blast command-line argument. The BLASTp search data, along with all gene models and gene model location datasets combined, are used as input for PanOCT. For a pan-genome dataset of syntenic ortholog clusters as constructed by Pangloss, clusters that contain an ortholog from all input genomes are classified as “core” clusters (containing “core” gene models) and clusters missing an ortholog from ≥1 input genomes are classified as “accessory” clusters (containing “accessory” gene models) [11]. Pangloss also generates nucleotide and amino acid datasets for every core and accessory cluster for further downstream analyses.

#### 2.1.3. Refinement of Pan-Genome Construction Based on Reciprocal Sequence Similarity

After construction of the initial pan-genome, the user has the option of refining the pan-genome with Pangloss via the --refine command-line argument (Figure 1). This method attempts to refine the PanOCT-derived microsyntenic pan-genome by accounting for microsynteny loss due to genome assembly artefacts or genomic rearrangements. In this method, Pangloss first extracts all accessory clusters from the accessory genome and parses the previously-generated all-vs.-all BLASTp data used for PanOCT. For each accessory cluster ***A***, Pangloss extracts the BLASTp data for each ortholog in ***A*** and generates a list of BLASTp top-hits to each strain genome not represented in ***A*** with ≥30% sequence identity. If this list matches another accessory cluster ***B*** in the accessory genome, Pangloss will then check if each ortholog in ***B*** has a reciprocal strain top-hit to each ortholog in ***A***. If ***A*** and ***B*** satisfy this criterion they are merged into a new cluster ***AB***, and ***A*** and ***B*** themselves are subsequently removed from the accessory genome. If this new cluster ***AB*** has an ortholog from every input strain genome in the dataset it is then reclassified as a core cluster [11].

#### 2.1.4. Functional Annotation and Characterisation of Pan-Genome Components

There are optional arguments in Pangloss through which the user can characterise pan-genomes once they are constructed (Figure 1). If InterProScan (https://www.ebi.ac.uk/interpro/download.html) is installed, the user can select to have the entire pan-genome dataset annotated with Pfam, InterPro and gene ontology (GO) information via the --ips command-line argument [44]. Additionally, if GOAtools (https://github.com/tanghaibao/goatools) is installed, the output from InterProScan can be used to perform GO-enrichment analysis of the core and accessory components of the pan-genome via the --go command-line argument, using Fischer’s exact test (FET) with parent term propagation and false discovery rate correction (*p* < 0.05) using a *p*-value distribution generated from 500 resampled *p*-values [45,48].

#### 2.1.5. Selection Analysis of Pan-Genome Using yn00

The user can perform selection analysis on core and accessory gene model clusters using yn00 from the PAML (http://abacus.gene.ucl.ac.uk/software/paml.html#download) package of phylogenetic software (enabled via the --yn00 command-line argument) (Figure 1) [43]. For each cluster in a pangenome dataset, an amino acid alignment is performed using MUSCLE (https://www.ebi.ac.uk/Tools/msa/muscle/) with the default parameters. A corresponding nucleotide alignment is then generated by Pangloss by transferring gaps in the amino acid alignment into the nucleotide data for the same cluster. yn00 selection analysis is handled by Biopython’s Bio.Phylo.PAML module (https://biopython.org/) and is run with the default parameters (universal genetic code, equal weighting of pathways between codons and estimated codon frequencies). From each cluster alignment, Pangloss will report where available the estimated transition/transversion rate ratio of the cluster (κ) and the number of pairwise alignments within the cluster that show evidence of positive selection according to Yang and Nielsen’s method where the d_N_/d_S_ ratio (ω) is ≥ 1, if ω ≠ ∞ [49].

#### 2.1.6. Visualisation of Pan-Genome Data

A number of optional methods of visualising pan-genome data are incorporated into Pangloss (Figure 1). A simple ring chart of the proportion of core and accessory gene models in a pangenome dataset is generated in R using the --size command-line argument. The same flag also generates a bar chart for the distribution of syntenic cluster sizes within a pangenome dataset and estimates the true size of the pan-genome using the Chao lower bound method in R, as previously implemented in the prokaryote pan-genome analysis package micropan [50,51]. The Chao lower bound method estimates the size of a population given a set of occurrence data for that population from singleton and doubleton occurrences [50]. In the case of pan-genomic data we can estimate the true number of syntenic clusters within a pan-genome ($\hat{N}$) given the observed number of clusters (*N*) from the numbers of 1-member and 2-member clusters in the pan-genome (*y_1_* and *y_2_*, respectively), as given by the equation [50]:

$$\hat{N}=N+\;\frac{y_{1}^{2}}{2y_{2}}$$

The Chao lower bound method is a conservative method of estimating true pan-genome size, but it is worth noting that this estimation may be skewed in cases of overabundance of singleton data (e.g., singleton genes arising from highly fragmented genomes) [51,52]. The distribution of syntenic orthologous gene models within the species accessory genome can be visualised using the R package UpSetR via the --upset command-line argument [35]. This generates an ortholog distribution plot based on the UpSet technique of visualising intersections of sets and their occurrences within a dataset using matrix representation, allowing for more input sets than similar Venn-based or Euler-based methods [53]. Finally, karyotype plots of the genomic locations of core and accessory gene models along each chromosome/contig within a genome, coloured by either pan-genome component or by syntenic cluster size, can be generated for each genome in a dataset using the Bioconductor package KaryoploteR (https://bioconductor.org/packages/release/bioc/html/karyoploteR.html) via the --karyo command-line argument [36,37].

### 2.2. Dataset Assembly

#### 2.2.1. *Yarrowia lipolytica*

Nuclear genome assembly data for seven *Yarrowia lipolytica* strains was obtained from GenBank. Each strain genome was selected based on geographic and environmental distribution, information on which is found in Table S1 [24,54,55,56]. Gene model and gene model location prediction was carried out for all *Y. lipolytica* strain genomes using Pangloss (Figure 1). GeneMark-ES gene model prediction was performed with a fungal branching point model and TransDecoder gene model prediction was performed with an amino acid sequence length cut-off of ≥200 aa. All predicted gene model sets were filtered against a set of 936 known pseudogenes or dubious open reading frames (ORFs) from *Saccharomyces cerevisiae* and *Candida albicans* obtained from the *Saccharomyces* and *Candida* Genome Database websites respectively, with a BLASTp e-value cut-off of 10^−4^ [47,57]. Gene models with sequence coverage of ≥70% to a pseudogene/dubious ORF were removed from the dataset (Table S1). BUSCO analysis for each strain gene model set was performed using the Saccharomycetales dataset (Table S1). In total, 45,533 gene models were predicted across our entire *Y. lipolytica* pan-genome dataset, with an average of 6504 gene models per strain and BUSCO completeness per gene model set ranging from approximately 83–89% (87.9% average) (Table S1).

#### 2.2.2. *Aspergillus fumigatus*

Nuclear genome assembly data for 12 *Aspergillus fumigatus* strains was obtained from GenBank. Each strain genome was previously used to construct an initial *A. fumigatus* species pan-genome using a similar approach to that implemented in Pangloss, and strains were selected based on geographic and environmental distribution including both clinical and wild-type strains [11] (Table S1). Gene model and gene model location prediction was carried out for all *A. fumigatus* genomes using Pangloss (Figure 1). GeneMark-ES gene model prediction was performed with a fungal branching point model and TransDecoder gene model prediction was performed with an amino acid sequence length cut-off of ≥200 aa. No filtering for pseudogenes or dubious ORFs was performed for the *A. fumigatus* dataset as no such data is available. BUSCO analysis for each strain gene model set was performed using the Eurotiomycetes dataset (Table S1). In total, 113,414 gene models were predicted across our entire *A. fumigatus* pan-genome dataset, with an average of 9451 gene models per strain and BUSCO completeness per gene model set ranging from approximately 93–97% (96% average) (Table S1).

### 2.3. Pangenome Analysis

#### 2.3.1. *Yarrowia lipolytica*

**Figure 2:**
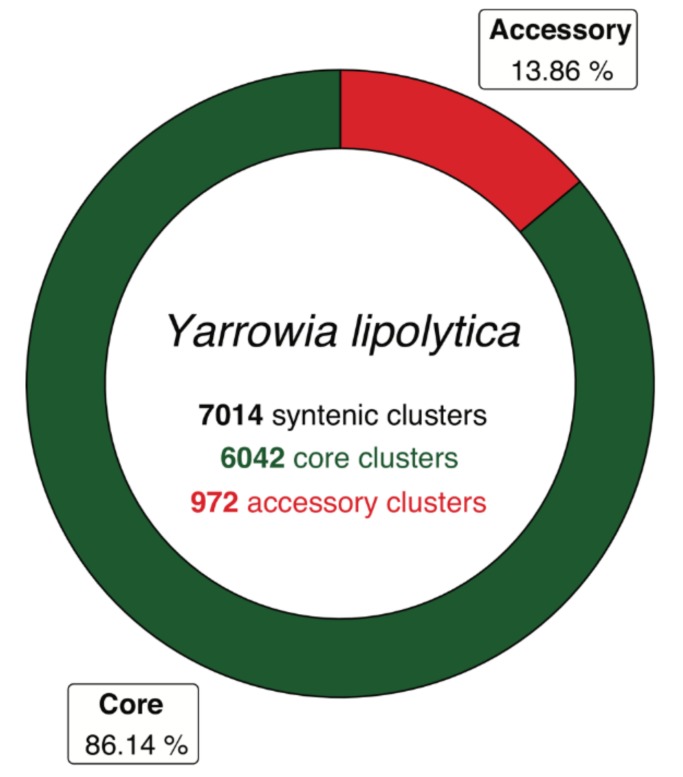
Pan-genome of *Yarrowia lipolytica* represented as a ring chart of proportions of core and accessory ortholog clusters within the total dataset. Modified from original figure generated by Pangloss. Core proportions coloured in green, accessory proportions coloured in red.

**Figure 3:**
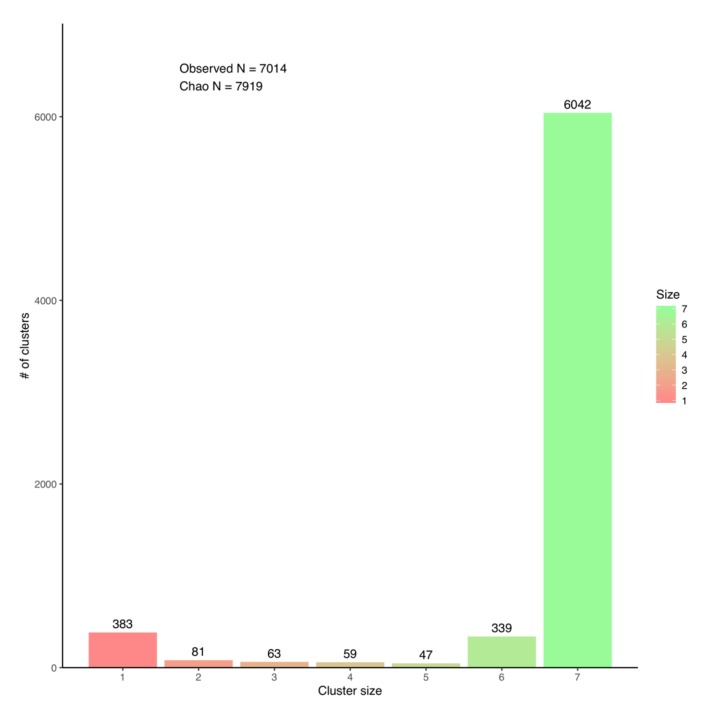
Bar chart representing the distribution of syntenic cluster sizes within *Yarrowia lipolytica* pan-genome and Chao’s lower bound estimation of true pan-genome size. Figure generated by Pangloss.

**Figure 4:**
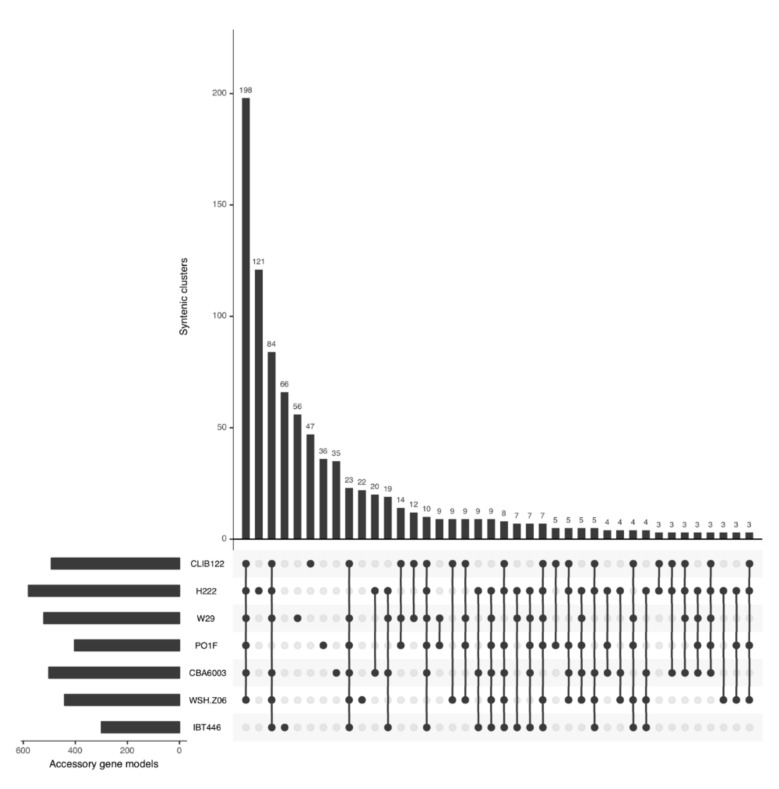
UpSet plot of the distribution of syntenic orthologs within the *Yarrowia lipolytica* accessory genome, ranked by syntenic cluster frequency. UpSet plots represent intersections between sets within data as a matrix and the number of occurrences of those intersections as a bar chart. In our case, the set intersection matrix represents clusters which contain a syntenic ortholog from 1–6 strains in our dataset and the number of their occurrences is given by the bar chart. Numbers of singleton clusters range from 22 in WSH-Z06 to 121 in H222. Figure generated by Pangloss.

**Figure 5:**
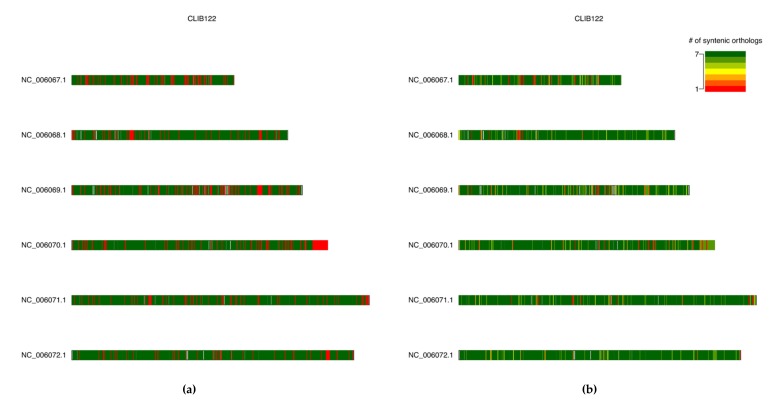
Karyotype plots of core and accessory gene model locations across the six chromosomes of *Yarrowia lipolytica* strain CLIB122. Left: (**a**) Gene model locations coloured by source pan-genome component (core: green, accessory: red). Right: (**b**) Gene model locations coloured by the size of their source syntenic cluster. Non-coding regions coloured in grey. Both figures generated by Pangloss.

An all-vs.-all BLASTp search for the entire *Y. lipolytica* dataset was performed within Pangloss with an e-value cut-off of 10^−4^. PanOCT analysis for the *Y. lipolytica* dataset was performed within Pangloss using the default parameters for PanOCT (CGN window = 5, sequence identity cut-off ≥35%). Pan-genome refinement was carried out within Pangloss (Table S1). Pfam, InterPro and gene ontology annotation of the dataset was performed using InterProScan with the default parameters [44,58,59,60]. GO-slim enrichment analysis was carried out for both the core and accessory *Y. lipolytica* genomes using GOATools. GO terms were mapped to the general GO-slim term basket and a Fischer’s exact test (FET) analysis with parent term propagation and false discovery rate (FDR) correction (*p* < 0.05) with a *p*-value distribution generated from 500 resampled *p*-values [45,48,60]. yn00 analysis of the *Y. lipolytica* pan-genome dataset was performed within Pangloss with the default parameters [43,49]. All plots were generated within Pangloss using its various R components as detailed above (Figure 1, Figure 2, Figure 3, Figure 4 and Figure 5).

#### 2.3.2. *Aspergillus fumigatus*

An all-vs.-all BLASTp search for the entire *A. fumigatus* dataset was performed within Pangloss with an e-value cut-off of 10^−4^. PanOCT analysis for the *A. fumigatus* dataset was performed within Pangloss using the default parameters for PanOCT (CGN window = 5, sequence identity cut-off ≥35%). Pan-genome refinement was carried out within Pangloss (Table S1).

## 3. Results

### 3.1. Analysis of the Yarrowia lipolytica Pan-Genome

A *Y. lipolytica* species pan-genome was constructed with Pangloss via PanOCT using publicly-available assembly data from seven strains, including the reference CLIB122 strain and a number of other industrially-relevant strains [24,54,55,56] (Table S1). Strain genomes ranged in size from 19.7–21.3 Mb, and the majority had been assembled to near-scaffold quality (Table S1). A total of 45,533 valid *Y. lipolytica* gene models were predicted by Pangloss after filtering for known pseudogenes from model yeasts, for an average of ~6505 gene models per strain genome (Table S1). Pangloss constructed a refined species pan-genome for *Y. lipolytica* containing 6042 core syntenic clusters (42,294 gene models in total) and 972 accessory syntenic clusters (3239 gene models in total) (Figure 2, Table 2 and Table S1). This gives a core:accessory proportion split of approximately 92:8 in terms of gene models and 87:13 in terms of unique syntenic clusters (Figure 2, Table S1). These core:accessory proportions were similar to our previous analyses of other yeasts such as *Saccharomyces cerevisiae* (85:15) and *Candida albicans* (91:9) [11]. Accessory genome size in individual *Y. lipolytica* strains varied from 303 gene models in IBT446 to 583 gene models in H222 (Table S1). Using Chao’s lower bound method, the size of the *Y. lipolytica* pan-genome was estimated to contain 7970 syntenic clusters (Figure 3). 341 syntenic clusters were missing an ortholog in one strain, with 202 clusters missing an ortholog from IBT446 only, and 390 syntenic clusters consisted of a singleton gene model (Figure 3 and Figure 4). The number of singleton gene models in individual strains varied from 23 gene models in WSH-Z06 and CBA6003 to 121 gene models in H222 (Figure 4). Karyotype plots were generated for each *Y. lipolytica* strain in our dataset and display varying amounts of accessory gene models distributed across the six chromosomes of *Y. lipolytica* (e.g., CLIB122 in Figure 5a,b). This is similar to our previous observation of accessory genome distribution within the *Candida albicans* pan-genome, which may have arisen due to a lack of non-clinical strain genomes for that species [11]. A large accessory region in chromosome D in CLIB122 (NC_006070.1, Figure 5a,b) appears to be the result of a gapped region in the same chromosome in PO1f, presumably arising from sequencing artefacts (Figure 5a,b).

### 3.2. Characterisation of the Yarrowia lipolytica Pan-Genome

Selection analysis was performed for all non-singleton clusters in the *Y. lipolytica* core and accessory genome using yn00, which estimates synonymous and non-synonymous rates of substitution within a gene family using pairwise comparisons [43]. Of the 6042 core clusters in the *Y. lipolytica* pan-genome dataset, 453 clusters had at least one pairwise alignment which had ω ≥ 1 (7% of all core clusters), whereas for the 582 non-singleton accessory clusters only 52 clusters had at least one pairwise alignment with ω ≥ 1 (9% of all non-singleton accessory clusters). It is possible that the low levels of positive selection (i.e., clusters with ≥1 pairwise alignment with ω ≥ 1) within the accessory genome reflects the potential lack of evolutionary distance between the strains in our *Y. lipolytica* dataset. The *Y. lipolytica* pangenome dataset was annotated with Pfam, InterPro and gene ontology data using InterProScan [44,58,59,60]. Approximately 77% of the total dataset (35,139 gene models) contained at least one Pfam domain. GO-slim enrichment analysis was performed for both core and accessory genomes using GOATools with the default parameters as implemented in Pangloss (Table S2). Unlike our previous analysis of term enrichment in fungal pan-genomes, transport processes appear to be enriched within the core *Y. lipolytica* genome and processes relating to the production of organic and aromatic compounds are enriched within the accessory *Y. lipolytica* genome (Table S2) [11]. The former may be due to the array of the lipid transport systems that *Y. lipolytica* uses to live in environments rich in hydrophobic substrates [61]. Similarly, genes whose functions are related to intracellular organelle function are enriched in the *Y. lipolytica* core genome—this may encompass the accumulation of lipids and fatty acids within organelles and lipid body formation within the *Y. lipolytica* cell (Table S2) [62].

### 3.3. Reanalysis of the Aspergillus fumigatus Pan-Genome

As a way of assessing the quality of Pangloss’s pan-genome construction we also reconstructed a species pan-genome for *Aspergillus fumigatus*, the opportunistic agent of invasive aspergillosis, using a previously-analysed dataset containing both clinical and wild-type strains [11,63] (Table 2, Table S1). A total of 113,414 valid *A. fumigatus* gene models were predicted by Pangloss with an average of ~9451 gene models per strain genome (Table 2, Table S1). Pangloss constructed a refined species pan-genome for *A. fumigatus* containing 7668 core syntenic clusters (92,016 gene models in total) and 1783 accessory syntenic clusters (21,398 gene models in total) (Table 2, Table S1). This gives a core:accessory proportion split of approximately 81:19 in terms of gene models and 67:33 in terms of unique syntenic clusters (Table 2, Table S1). These core:accessory proportions are relatively in line with our previous study of the same *A. fumigatus* pan-genome dataset, which found core:accessory proportion splits of 83:17 in terms of gene models and 73:27 in terms of unique syntenic clusters [11]. Variation between the two *A. fumigatus* pan-genome analyses is a result of performing gene prediction using Exonerate in our initial analysis but not in this subsequent reanalysis [11].

## 4. Discussion

As pan-genome analysis of eukaryotes becomes more commonplace, ideally the amount of software to construct and characterise eukaryote pan-genome should begin to match that which is already available for prokaryotes. Our software pipeline Pangloss applies a sequence similarity and synteny-based approach from prokaryote pan-genome analysis, implemented in the previously-published Perl software PanOCT, to eukaryote pan-genome analysis and allows the user to perform their own gene prediction and downstream characterisation and visualisation of pan-genome data from one self-contained script [11,22]. Although our pipeline has been designed for eukaryote pan-genome analysis, as PanOCT is a prokaryote method in origin, Pangloss should also support prokaryote datasets—albeit with some modifications to gene model prediction strategies by the user. Unlike other common gene clustering approaches, such as MCL, PanOCT incorporates local synteny via assessing the CGN between potential orthologs as a criterion to clustering in addition to sequence similarity [19,22]. This makes PanOCT distinct from most clustering approaches in that it can distinguish orthologs from paralogs (i.e., if one assumes that “true” orthologs are more likely to be located in relatively-similar regions of their respective genomes they then should in turn be more likely to cluster together when syntenic conservation is taken into consideration). This is of particular relevance to eukaryote pan-genomes, as gene duplication plays a substantial role in eukaryote gene family and genome evolution [11,64]. Although this approach is more stringent than clustering gene families based on approaches like MCL or BLAST searches alone, it is potentially more reflective of evolution on a gene-level basis within strains of the same species.

There are ways in which our approach can be improved upon in future methodologies, both in terms of prediction and analytic strategies. For example, Pangloss has an optional Exonerate-based gene model prediction strategy which searches input genomes for translated homologs of reference sequences [33]. This is an exhaustive approach that may pick up potential gene models missed by GeneMark-ES and/or TransDecoder, but it is also time-inefficient. To search all 6472 reference protein sequences from *Y. lipolytica* CLIB222 against a single *Y. lipolytica* genome takes, on average, four hours on three threads on a server running Ubuntu 18.04.2 LTS (approximately nine sequences per minute per thread), whereas both GeneMark-ES gene model prediction with fungal point branching and subsequent ORF prediction in non-coding regions with TransDecoder performed on the same genome with the same number of threads typically takes ~30–35 min. It is for this reason primarily that we have made the Exonerate-based strategy optional for any gene prediction that is performed by Pangloss. Furthermore, PanOCT’s memory usage increases exponentially per strain added, notwithstanding the potentially complex distribution of gene models between strains themselves [11,22]. Constructing a species pan-genome using PanOCT from a small and relatively well-conserved dataset, such as that for our *Y. lipolytica* or *A. fumigatus* studies, should be achievable on most standard hardware. For larger datasets, such as our previous pan-genome analysis of 100 *Saccharomyces cerevisiae* genomes; however, it may be preferable to perform such analysis on a high-performance computational environment or otherwise an alternative synteny-based method of pan-genome construction may be more appropriate [11]. Finally, we would encourage users to interrogate and visualise the results of analysis using Pangloss and adjust the input parameters where appropriate for their data. In our case, the parameters which were chosen for use in Pangloss for this analysis (e.g., BLAST e-value cut-off, CGN window size) are largely based on those from our previous analysis of fungal pan-genomes or other studies using PanOCT [11,22]. Depending on the size of a pan-genome dataset or the species of interest, different cut-offs may be more suitable (e.g., for species with longer average gene lengths a lower sequence identity cut-off for PanOCT clustering than the default (>35%) may be more appropriate). Many of these parameters can be adjusted in the configuration file provided with Pangloss.

## 5. Conclusions

Pan-genome analysis of eukaryotes has become more common, but many of the available software for pan-genome analysis are intended for use with prokaryote data. We have developed Pangloss, a pipeline that allows users to generate input data and construct species pan-genomes for microbial eukaryotes using the synteny-dependent PanOCT method and various downstream characterisation analyses. To demonstrate the capabilities of our pipeline we constructed a species pan-genome for *Yarrowia lipolytica*, an oleaginous yeast with potential biotechnological applications, and performed various functional and data visualisation analyses using Pangloss. The *Y. lipolytica* pangenome is similar in terms of core and accessory genome proportions to previously analysed fungal pan-genomes but is unique in that biological processes such as transport are statistically-enriched in the core genome. We also used Pangloss to reconstruct a species pan-genome for the respiratory pathogen *Aspergillus fumigatus* using a previously-analysed dataset and found that Pangloss generated a similar pan-genomic structure for *A. fumigatus* to that of our previous analysis. Building on our previous work on fungal pan-genomes, this study not only provides further evidence for pan-genomic structure within eukaryote species but also presents a methodological pipeline for future eukaryote pan-genome analysis.

## Figures and Tables

**Table 1:** List of various dependencies for Pangloss, versions tested in parentheses. PanOCT included with Pangloss. See http://github.com/chmccarthy/Pangloss/ for download location and detailed installation instructions for each dependency.

Dependencies	Function
Python (2.7.10) *. BioPython (1.7.3) [32]	Base environment for Pangloss.
Exonerate (2.4) [33], GeneMark-ES (4.3.8) [38], TransDecoder (5.5) [39]	Gene model prediction.
BLAST+ (2.9.0) [40]	All-vs.-all sequence similarity search, dubious gene similarity search.
BUSCO (3.1) [41]	Gene model set completeness analysis.
PanOCT (3.2) [22]	Pan-genome construction.
MUSCLE (3.8.31) [42], PAML (4.8) [43]	Selection analysis of core/accessory cluster alignment using yn00.
InterProScan (5.34)^†^ [44], GOATools (0.8.12) [45]	Functional classification and functional enrichment analysis of pan-genome.
R (3.6), ggplot (3.2) [34], ggrepel (0.8.1), UpSetR (1.4) [35], Bioconductor (3.9) [36], KaryoploteR (1.10.3) [37]	Visualisation of pan-genome size and distributions across genomes.

* Required for all analyses. ^†^ InterProScan is only available for Linux distributions.

**Table 2:** Pan-genomes of *Yarrowia lipolytica* and *Aspergillus fumigatus*. Refer to Table S1 for further information including strain assembly statistics, BUSCO completeness and links to relevant literature.

Species	Strains	Core Genome		Accessory Genome		Pan-Genome	
		Gene Models	Clusters	Gene Models	Clusters	Gene Models	Clusters
*Yarrowia lipolytica*	7	42,294	6042	3239	972	45,533	7014
*Aspergillus fumigatus*	12	92,016	7668	21,398	3727	113,414	11,395

## Supplementary Materials

The following are available online at https://www.mdpi.com/2073-4425/10/7/521/s1. Table S1, Information for *Yarrowia lipolytica* and *Aspergillus fumigatus* pan-genome datasets. Core gene models labelled in green, accessory gene models labelled in red. References and strain information taken from cited articles where available, otherwise from GenBank or similar resources with relevant links included. Table S2. GO-slim enrichment analysis for the *Yarrowia lipolytica* pan-genome dataset. Fischer’s exact test with FDR correction (*p* < 0.05) carried out using GOATools within Pangloss. All terms present in the table are either significantly over- or under-represented in either the *Y. lipolytica* core or accessory genome. Significantly over-represented terms labelled green, significantly under-represented terms labelled red.
